# Comparison Between a Minimally Invasive Capillary Blood Sampling Technique and Venipuncture for Core Vaccine Antibody Titration in Dogs

**DOI:** 10.3390/vaccines14050427

**Published:** 2026-05-10

**Authors:** Paola Dall‘Ara, Lucia Tamanza, Federico Alghisi, Davide Raccagni, Alice Garegnani, Joel Filipe

**Affiliations:** 1 Department of Veterinary Medicine and Animal Sciences (DIVAS), University of Milan, Via dell’Università 6, 26900 Lodi, LO, Italy; alice.garegnani@unimi.it (A.G.); joel.soares@unimi.it (J.F.); 2 Clinica Veterinaria La Meridiana, Via Torre Calini Carabbiolo, 1, 25030 Maclodio, BS, Italy; lucia.tamanza.vet@outlook.it (L.T.); info@cvlameridiana.it (F.A.); davide.raccagni@unimi.it (D.R.)

**Keywords:** dog, core vaccines, antibody titration, point-of-care testing, capillary blood sampling, minimally invasive technique, protection evaluation

## Abstract

**Background/Objectives**: International guidelines advocate for personalized vaccination protocols using point-of-care (POC) antibody titration to identify dogs requiring boosters for CPV-2, CDV, and CAdV-1. As traditional venipuncture can be challenging in specific patients, this study evaluate the clinical agreement of a novel minimally invasive capillary blood sampling technique (ear-prick) for core vaccine antibody titration. **Methods**: Paired blood samples were collected from 55 healthy dogs using venipuncture and an ear-prick technique with a portable lancet. Antibody titers were determined using a semi-quantitative POC kit (VacciCheck^®^ Canine). The procedure was optimized comparing 28G and 21G lancets, with the latter used in 43 dogs to ensure adequate blood flow. Comprehensive statistical methods evaluated the correlation and agreement between the two sampling techniques. **Results**: Statistical analysis showed no significant differences between sampling methods (*p* > 0.05). In the optimized group (21G lancet), full match rates reached 81.4% for CPV-2, 76.8% for CDV, and 74.4% for CAdV-1. Clinical concordance was exceptionally high: 95.3% for CPV-2, 90.7% for CDV, and 100% for CAdV-1. Statistical analysis confirmed perfect agreement (1.00) for CPV-2 and CAdV-1, and moderate agreement (0.48) for CDV. **Conclusions**: The ear-prick technique using a 21G lancet is a reliable, minimally invasive alternative to venipuncture for antibody titration. This method simplifies clinical procedures and facilitates personalized immunization monitoring. Given the minimal blood volume required, it represents a versatile approach for evaluating immune status and protection levels to core vaccines in diverse settings, including pediatric and shelter medicine.

## 1. Introduction

Vaccination remains the cornerstone of preventing severe and widespread infectious diseases in both human and veterinary medicine. According to the World Small Animal Veterinary Association (WSAVA) guidelines [1], the American Animal Hospital Association (AAHA) guidelines [2] and other leading international veterinary organizations and experts [3,4,5,6], vaccines for companion animals are categorized into core and non-core. Core vaccines are considered essential for all dogs to protect against life-threatening and highly contagious diseases. Non-core vaccines are recommended based on individual risk assessments and lifestyle. Among the most significant threats to the canine population, Canine Parvovirus type 2 (CPV-2) remains one of the primary concerns. It is responsible for severe hemorrhagic gastroenteritis and continues to cause high mortality rates, particularly in puppies and unprotected individuals, despite the widespread availability of effective vaccines [7,8,9,10,11,12,13,14,15]. Similarly, Canine Distemper Virus (CDV) and Canine Adenovirus type 1 (CAdV-1) represent substantial multisystemic risks. CDV is characterized by its high contagiousness and a complex clinical course involving respiratory, gastrointestinal, and often fatal neurological signs in susceptible dogs. Although its prevalence has decreased in many regions due to successful immunization programs, sporadic outbreaks continue to occur, especially in high-density or poorly vaccinated populations [16,17,18,19,20,21]. On the other hand, CAdV-1 is the causative agent of Infectious Canine Hepatitis (ICH), a severe disease that can lead to acute hepatic failure, vascular damage, and sudden death [22,23,24,25,26].

Given the severity of these core pathogens, ensuring a robust and long-lasting immune response is not only a clinical priority for the individual patient but also a fundamental pillar of collective disease control and public health.

Modern preventive protocols have shifted away from traditional annual revaccination. Current immunological evidence demonstrates that modified live vaccines (MLVs) provide protection lasting at least three years, often extending throughout the animal’s lifetime [1,2,6,27,28,29]. However, vaccine-induced immunity is not universally uniform. Several factors can significantly compromise immune efficacy, such as maternally derived antibody (MDA) interference in puppies [6,11,30,31,32,33,34,35,36,37], the progressive immunosenescence in elderly patients [38,39,40,41,42], and the occurrence of genetic “non-responders” within certain breeds [1,6,10,43,44]. Furthermore, individual host factors, including inadequate nutritional status, poor health status or underlying metabolic stress, can further impair the ability to mount or maintain a protective immunological memory [6,7,10,34,45,46,47].

Consequently, contemporary veterinary medicine advocates for the personalization of vaccination protocols, shifting toward an individualized patient approach. In this context, the use of point-of-care (POC) rapid kits for core vaccine antibody titration is becoming a preferred practice, allowing for the precise identification of dogs requiring a booster [1,6,48,49,50,51,52].

However, obtaining the blood samples necessary for these tests can present significant clinical challenges. Factors such as young age (puppies), extremely small body size, or behavioral issues, including anxiety or aggression, can complicate traditional venipuncture. To date, standardized minimally invasive sampling techniques for routine clinical application remain limited.

The adoption of minimally invasive blood sampling techniques represents an opportunity to extend antibody titration to previously overlooked clinical scenarios. Traditional venipuncture can be highly stressful for highly nervous, agitated or aggressive patients, potentially leading to vasovagal syncope and compromising the veterinarian-client-patient bond. In contrast, the use of micro-sampling allows for rapid collection with minimal discomfort, preserving positive compliance for future visits. Furthermore, this methodology is particularly valuable in pediatric medicine for monitoring maternally derived antibodies (MDA) and confirming post-vaccination immunity, allowing for precise immunization scheduling without the need for frequent, invasive blood draws.

The aim of this study was to design and evaluate the clinical agreement of a novel minimally invasive blood sampling technique specifically tailored for diagnostic tests requiring micro-volumes of blood, such as core vaccine antibody titration tests.

## 2. Materials and Methods

### 2.1. Study Population and Ethical Statement

Blood samples were collected from 55 dogs over a one-year period (September 2024 to September 2025) for antibody titration and other diagnostic purposes. The 55 dogs were selected using a purposive sampling approach to ensure a representative diversity of clinical presentations. Criteria for inclusion focused on maximizing variability in body size (ranging from toy to giant breeds), age, and physical characteristics that could influence ear-prick feasibility. Specifically, the cohort included dogs with different ear conformations (erect vs. pendulous) and various coat types (short, long, and wire-haired) to simulate the diverse population typically encountered in field veterinary practice.

The study protocol was approved by the Ethical Committee of the University of Milan (OPBA_120_2024). In accordance with the institutional Ethical Committee’s guidelines, residual aliquots of samples collected with informed owner consent can be used for research purposes without additional formal authorization (EC decision 29 October 2012, renewed under protocol no. 02-2016).

### 2.2. Sampling Procedures

To compare the diagnostic agreement between different sampling methods, paired blood samples were collected from each dog using two distinct procedures. Traditional venous sampling was conducted via venipuncture of the cephalic or saphenous vein using standard techniques. Concurrently, a minimally invasive capillary sampling was performed on the inner surface of the pinna (ear-prick) using a portable lancet device, similar to those employed for blood glucose monitoring in diabetic patients.

Both the venous and capillary samples were analyzed to determine specific antibody titers against canine core pathogens, specifically Canine Parvovirus type 2 (CPV-2), Canine Distemper Virus (CDV), and Canine Adenovirus type 1 (CAdV-1), using the VacciCheck^®^ Canine (Biogal, Kibbutz Galed, Israel) point-of-care (POC) test. The results were then statistically analyzed to assess the degree of agreement between the two sampling techniques.

For each dog, a comprehensive clinical record was established using a unique identification number. This record included signalment (breed, body size, sex and age), sampling date, current health status, and complete vaccination history, ensuring a detailed clinical profile for all subjects.

### 2.3. Sample Collection and Processing

For each dog enrolled in the study, two separate blood samples were collected. First, a standard venous sample of 1 mL was obtained from the cephalic or saphenous vein. The blood was collected into heparinized tubes and centrifuged at 1500 rpm for 10 min to obtain approximately 0.5 mL of plasma. These samples were then transferred into labeled tubes identified by the dog’s progressive number followed by the letter “A” (e.g., 1A, 2A, etc.) to indicate the traditional sampling method.

**Figure 1:**
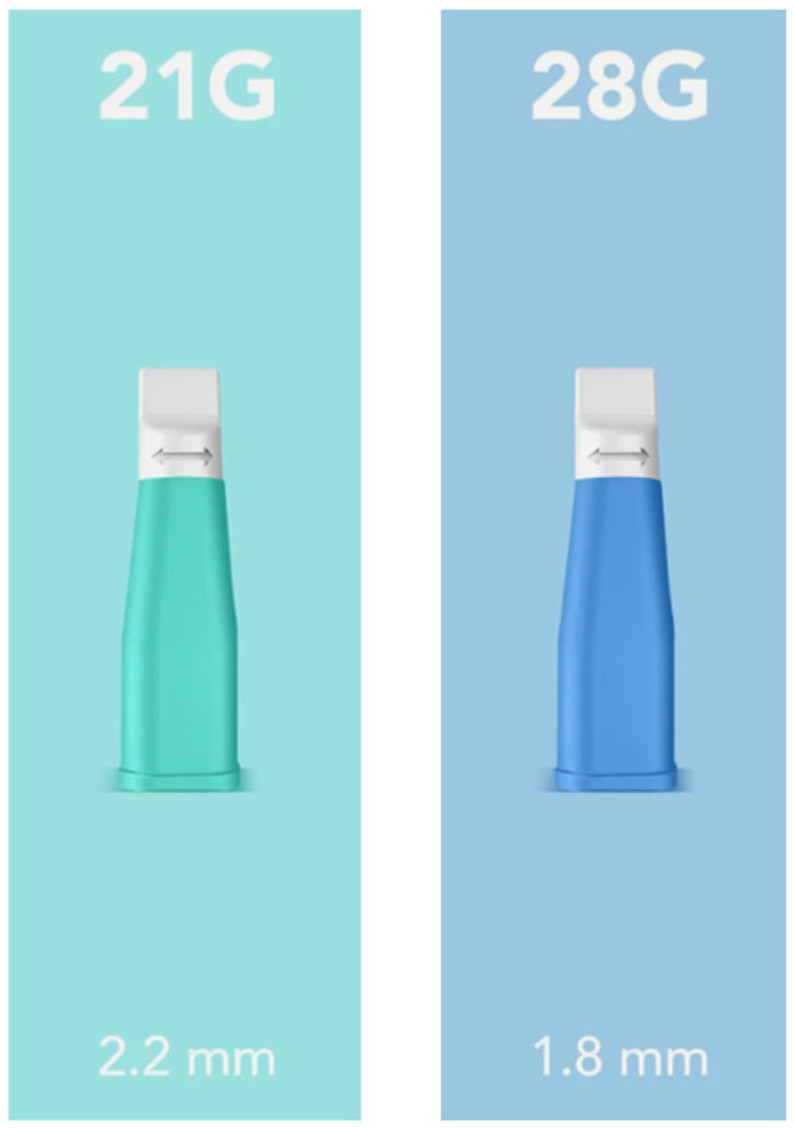
Safety lancets used for capillary blood sampling. The devices differ by needle gauge (G) and penetration depth (mm). In this study, the 21G and 28G models were compared to determine the most effective tool for blood collection from the canine pinna (Aiesi Hospital Service S.a.s, Naples, Italy).

Subsequently, a minimally invasive capillary sample was performed on the same animal. This second procedure was carried out using sterile, single-use safety lancets with a retractable needle system (Securlance, Aiesi Hospital Service S.a.s, Naples, Italy). The study followed an adaptive design; an initial pilot phase (*n* = 12) using 28G lancets has identified a need for larger capillary volumes, leading to the selection of 21G lancets for the subsequent exploratory cohort (*n* = 43) (Figure 1).

**Figure 2:**
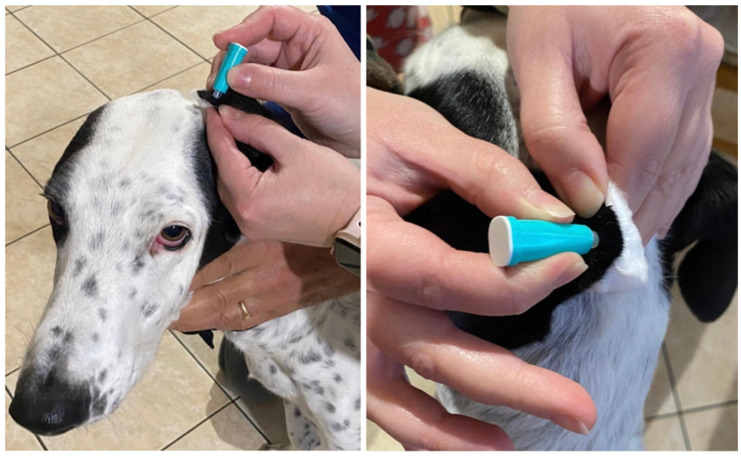
Capillary blood collection procedure from the inner surface of the canine pinna. The image illustrates the positioning of the safety lancet on the lateral margin (**left**) and the use of a cotton ball on the external surface to provide stability and counter-pressure during the puncture (**right**).

The capillary sampling procedure was performed on the inner surface of the pinna, targeting the most lateral margin. To optimize procedural speed, trichotomy was performed selectively, only when the hair length at the ear margin was sufficient to interfere with the formation of a ‘hanging drop’. To ensure a stable and controlled puncture, a cotton ball was frequently placed on the external surface of the ear to provide counter-pressure (Figure 2).

**Figure 3:**
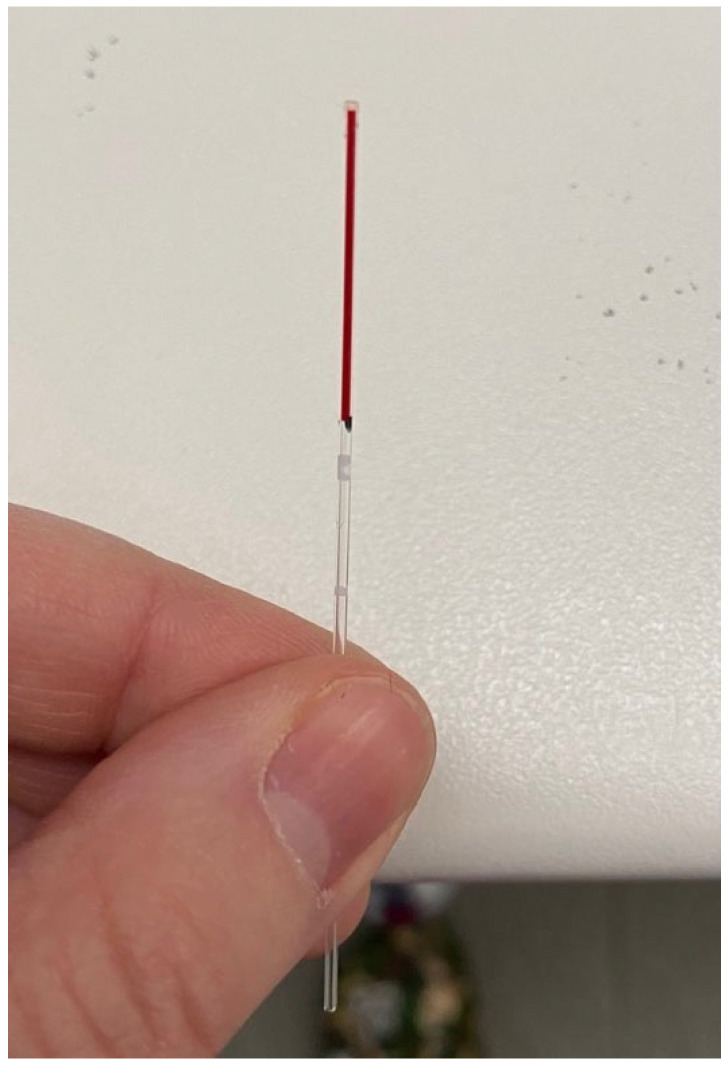
Collection of capillary blood into a glass tube via the ear-prick technique. The image demonstrates the precise micro-volume of blood obtained from the ear pinna margin, sufficient for direct sampling or subsequent dilution for point-of-care testing.

**Figure 4:**
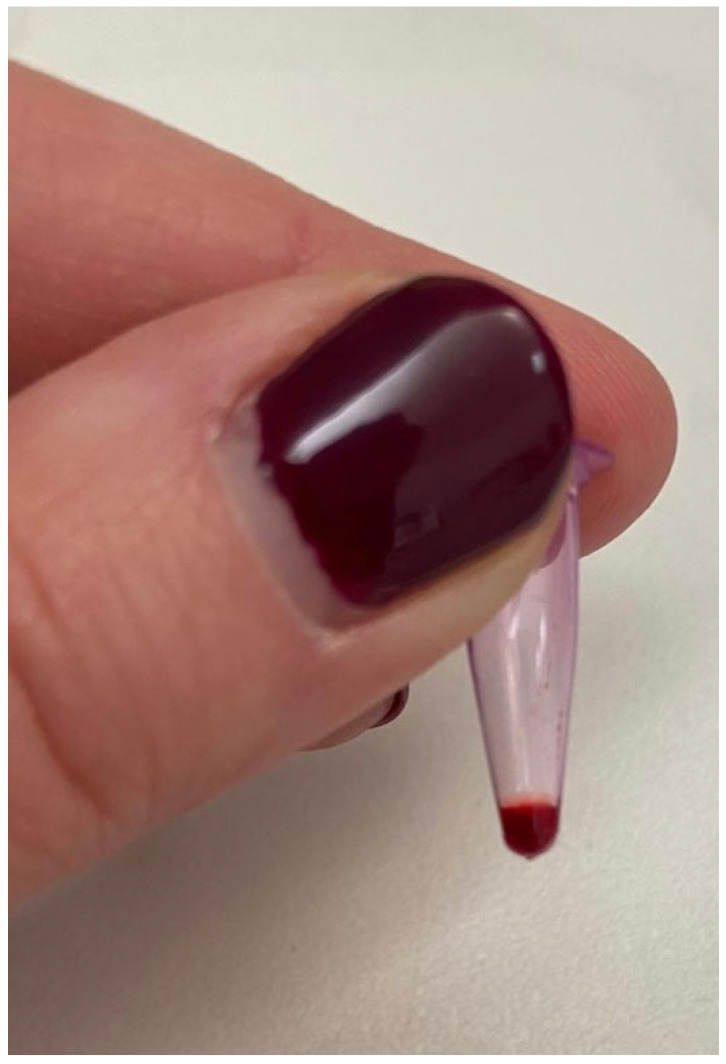
Capillary blood sample diluted 1:2 with sterile distilled water was transferred into a 0.2 mL Eppendorf tube. This preparation provides the necessary volume and fluidity for analysis with the point-of-care test.

Following the puncture, a small drop of blood was obtained through gentle digital expression of the surrounding pinna tissue. The blood was then collected using a glass capillary tube with a diameter of 0.9–1.1 mm (Figure 3). On the capillary tube a visual reference mark was used to ensure a 1:2 dilution by adding an equal volume of sterile distilled water. The diluted sample was then immediately transferred into 0.2 mL Eppendorf tubes. The use of the same glass capillary for both blood and diluent was intended to standardize the volumetric proportions as consistently as possible under clinical field conditions. This practical, field-adapted approach was designed to obtain a sufficient sample volume for POC testing and to prevent rapid coagulation (Figure 4).

### 2.4. Antibody Titration (VacciCheck^®^)

**Figure 5:**
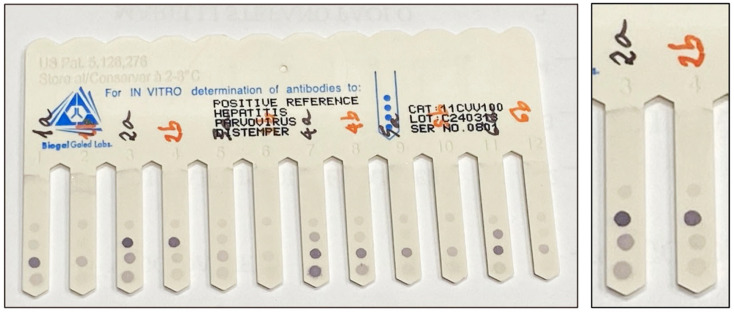
Representative VacciCheck^®^ Canine comb from the study. The complete comb shows multiple samples being processed (**left**), while the detailed view (**right**) highlights the adjacent results for a single dog (Sample no. 2), comparing the undiluted venous plasma (A) and the 1:2 diluted capillary whole blood (B) which must be multiplied by the dilution factor (2). This simultaneous processing allows for direct assessment of the two sampling techniques.

Antibody titers against CPV-2, CDV, and CAdV-1 were determined using a rapid semi-quantitative dot-ELISA-based kit (VacciCheck^®^ Canine; Biogal, Kibbutz Galed, Israel; supplied in Italy by Agrolabo, Scarmagno, Italy). This system has been extensively validated and utilized in numerous recent antibody titration studies [1,6,12,18,40,47,48,50,51,52,53], showing high sensitivity and specificity for all three pathogens. The two different sampling protocols were compared for each dog following the manufacturer’s instructions: 5 µL of undiluted venous plasma (Protocol A) and 10 µL of the 1:2 diluted capillary whole blood (Protocol B). It is important to note that the 1:2 dilution was specifically designed to ensure that Protocol B contained an equivalent amount of pure sample to that required by the manufacturer for whole blood analysis, while simultaneously providing the necessary volume and fluidity for proper processing. To ensure accurate interpretation, the semi-quantitative results obtained from Protocol B were multiplied by the dilution factor (2) to determine the final antibody titer, allowing for a direct comparison with the undiluted gold standard samples. To ensure direct comparability and minimize procedural variables, both samples from the same dog were processed simultaneously in adjacent teeth on the same comb (Figure 5).

The antibody concentration was determined by the color intensity of the resulting spots compared to a provided scale ranging from 0 to 6 “S” units. According to the manufacturer’s standards, an S value of 0 (S0) corresponds to antibody titers of <1:20 for CPV-2, <1:8 for CDV, and <1:4 for CAdV-1. An S value of 3 (S3) represents the threshold for a significant positive response, equivalent to titers of 1:80 for CPV-2, 1:32 for CDV, and 1:16 for CAdV-1. For the purpose of this study, antibody titers ≥ S3 were considered indicative of specific protection against these three diseases (Appendix A Table A1) and expressed as Protective Antibody Titers (PATs) [54]. To ensure accurate clinical interpretation, the semi-quantitative results obtained from the diluted capillary samples (Protocol B) were adjusted by the dilution factor (2) before being categorized according to these standardized S-unit thresholds.

### 2.5. Statistical Analysis

Statistical analysis was performed using Graph Pad Prism 8 software (GraphPad Software, La Jolla, CA, USA). Statistical analyses were performed using non-parametric methods due to the non-normal distribution of the data, as assessed by the Shapiro–Wilk test. Specifically, the Mann–Whitney U test was used for comparisons between independent groups, while the Wilcoxon signed-rank test was applied for paired data. Additionally, Cohen’s kappa coefficient was calculated to assess the agreement between the traditional blood sampling (A) and the ear-prick method (B). A significance threshold of *p* < 0.05 was adopted for all statistical tests. Due to the expected low prevalence of unprotected animals in the study population (a finding consistent with national epidemiological data [48]), diagnostic indices such as sensitivity, specificity, PPV, NPV and 95% confidence intervals were not calculated, as the very limited number of negative events would result in statistically unstable estimates.

### 2.6. Field Application: Seized Puppies in a Licensed Pet Shop Setting

The clinical feasibility of the ear-prick technique was applied in a real-world scenario. The field application was triggered by a health emergency within a licensed pet shop, where the death of a puppy due to Canine Parvovirus (CPV-2) led to a judicial seizure of the remaining 70 animals. Although these puppies were microchipped and possessed Pet passports indicating an age of 4–5 months, their physical development suggested they were significantly younger. Given their vulnerability—due to long-distance transport, potential early weaning, and overcrowding—the ear-prick micro-sampling method was selected to provide health authorities with reliable immunological data in the shortest possible time while minimizing animal distress. To manage the outbreak, all puppies underwent simultaneous fecal swab testing via in-clinic rapid tests for CPV-2 and blood sampling for antibody titration using the 1:2 dilution capillary method.

## 3. Results

### 3.1. Study Population

A total of 55 client-owned dogs were included in the study. Of these, 37 (67.3%) were females and 18 (32.7%) were males; since reproductive status (intact/neutered) was not always available, this information was omitted. Age ranged from 5 months to 14 years, categorized as follows: 3 puppies (5.5%), 26 adults (47.3%), 21 seniors (38.2%), and 5 geriatrics (9.0%). Breed distribution was diverse: 44 dogs were purebred (80.0%), while 11 were crossbred (20.0%). The most represented breed was the Golden Retriever (6, 10.9%), followed by the Gordon Setter (5, 9.0%), and both French and English Bulldogs (4 dogs each, 7.3%). Regarding size, 3 dogs were extra-small (<5 kg, 5.5%), 7 small (≥5–<10 kg, 12.7%), 19 medium (≥10–<25 kg, 34.5%), 25 large (≥25–<45 kg, 45.5%), and 1 giant (>45 kg, 1.8%). All dogs were clinically healthy. Finally, all dogs had received core vaccines at least once in their lifetime (ranging from 1.5 months to 8 years prior to sample collection). Dogs were grouped according to the time elapsed since their last core vaccination: <1 year (24, 43.6%), ≥1–<3 years (29, 52.7%), and ≥3 years (2, 3.7%).

### 3.2. Minimally Invasive Blood Sampling Comparison

Initially, blood collection was performed using a 28G lancet. However, it quickly became apparent that this gauge was insufficient to consistently obtain a blood drop of adequate volume for the study requirements. Consequently, after the first 12 samples (21.8%) collected with the 28G lancet, the procedure was optimized by switching to a larger 21G lancet for the remaining 43 samples (78.2%). The 21G gauge allowed for a more copious blood flow, ensuring an optimal sample volume for the subsequent analysis. Despite the rapid clotting time of canine capillary blood in non-heparinized tubes, all collected samples were successfully processed and titrated thanks to immediate dilution in distilled water. No subjects were excluded from the analysis due to technical sampling failures.

The correlation between the antibody titers of the two samples (A and B) obtained for each dog using the VacciCheck^®^ Canine test is summarized in Table 1. Results focus on Canine Parvovirus type 2 (CPV-2), Canine Distemper Virus (CDV), and Canine Infectious Hepatitis (CAdV-1) across the initial 55 dogs enrolled in the study.

The results showed a good, though not absolute, overlap between the antibody titers obtained from the two sampling methods (A and B) for all three pathogens. The highest full match was observed for CPV-2 (72.7%, 40/55), followed by CDV (70.9%, 39/55) and CAdV-1 (65.5%, 36/55). In no instance did the titers differ by only half a grade. However, a difference of exactly one titer (either higher or lower) was observed in several cases: 8 dogs (+1, 14.6%) and 4 dogs (−1, 7.3%) for CPV-2, 7 dogs (+1, 12.7%) and 5 dogs (−1, 9.1%) for CDV, and 11 dogs (+1, 20.0%) and 6 dogs (−1, 10.9%) for CAdV-1. Consequently, results were further aggregated into three concordance categories (good, poor, bad match) (Table 2).

The good match group (comprising identical results and those with a clinical variation of only ±1 titer) showed remarkably high percentages across all pathogens: 94.5% (52/55) for CPV-2, 92.7% (51/55) for CDV, and 96.4% (53/55) for CAdV-1. Interestingly, no dogs fell into the poor match group (≥1 ½ titer discrepancy). Major discrepancies, classified as a bad match (±2 titers or more discrepancy), were infrequent, occurring in only 5.5% of cases for CPV-2 (3/55), 7.3% for CDV (4/55), and 3.6% for CAdV-1 (2/55).

**Figure 6:**
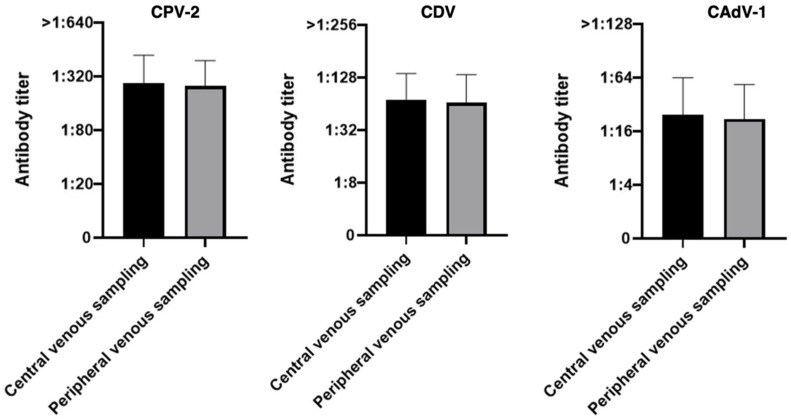
Antibody titers against Canine Parvovirus type 2 (CPV-2), Canine Distemper Virus (CDV), and Canine Adenovirus type 1 (CAdV-1) considering the two types of sampling methods in the 55 dogs (Wilcoxon signed-rank test).

Analyzing the group of 55 dogs, no statistically significant differences were observed between the two sampling methods for any of the pathogens analyzed (*p*-value 0.27 for CPV-2, 0.28 for CDV and 0.21 for CAdV-1), indicating that any differences observed between the two methods are likely attributable to chance rather than to a real effect of the sampling method (Figure 6).

On the same entire study population of 55 dogs, the concordance between the gold standard blood method and the ear-prick technique was evaluated using the Cohen’s kappa method (Appendix A Table A2). For CPV-2, the results demonstrated a perfect agreement, with a coefficient of 1.00 (*p* < 0.001), as both methods identified the same 54 protected and 1 unprotected dog. Regarding CAdV-1, the analysis showed an almost perfect agreement (kappa = 0.90), with an observed concordance of 96.3% (53/55). The slight discrepancy was due to two dogs identified as unprotected by the capillary method but protected by the serum test. For CDV, the agreement was classified as moderate (kappa = 0.47), despite a high observed concordance of 92.7% (51/55).

### 3.3. Capillary Sampling Device Comparison

In this study, two different types of lancets were evaluated based on their needle gauge (21G and 28G). As the gauge (G) number increases, the needle diameter decreases; consequently, 21G needles have a larger diameter compared to the 28G ones. The dogs were categorized based on the specific needle gauge used during the minimally invasive sampling procedure, as detailed in Table 3.

**Figure 7:**
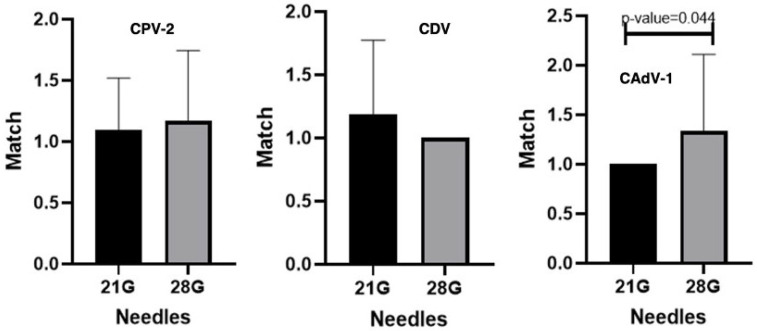
Antibody titers against Canine Parvovirus type 2 (CPV-2), Canine Distemper Virus (CDV), and Canine Adenovirus type 1 (CAdV-1) considering the two types of needles (21G vs. 28G, Mann–Whitney U test.

When comparing different needle types, no statistically significant differences were detected for CDV and CPV-2, indicating that the choice of needle does not affect antibody measurements for these pathogens. In contrast, a statistically significant difference was observed for CAdV-1 (*p* = 0.044), suggesting a potential influence of the sampling device on the measured antibody levels for this virus (Figure 7).

Initially, the study utilized 28G lancets almost exclusively. However, after the first few procedures, significant technical challenges were encountered, primarily related to insufficient blood flow. To address this, the protocol was optimized by switching to 21G lancets, which proved far more suitable for the purpose, consistently providing a more copious blood drop. Consequently, the 21G lancet became the standard device for the majority of the study (43 out of 55 dogs). To ensure the highest analytical reliability and minimize variables related to inadequate sample volume, the 12 samples collected with the 28G needle were excluded from the final analysis due to insufficient sample volume and inconsistent collection performance. Therefore, the subsequent results focus exclusively on the 43 samples obtained under optimal conditions using the 21G device (Table 4).

The exclusion of samples collected with the unsuitable 28G device led to a marked improvement in the overall results. Compared to the initial total cohort (*n* = 55), the refined group of 43 dogs sampled exclusively with the 21G lancet showed an increase in full match rates of nearly 10 percentage points for all three pathogens: CPV-2 (81.4% vs. 72.7%), CDV (76.8% vs. 69.0%), and CAdV-1 (74.4% vs. 65.5%). Consistent with the preliminary analysis, no discrepancies of half a titer or one-and-a-half titers were recorded in these optimized groups. In the remaining cases, the difference was limited to exactly one titer (higher or lower).

Consequently, as previously done with 55 dogs, all 43 samples with a full match or a maximum difference of ±1 titer were categorized into the good match group and compared against those with more discordant results (poor match or bad match), as summarized in Table 5.

**Figure 8:**
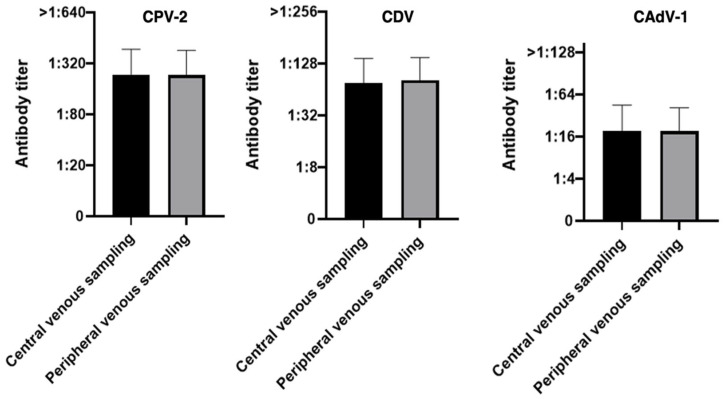
Antibody titers against Canine Parvovirus type 2 (CPV-2), Canine Distemper Virus (CDV), and Canine Adenovirus type 1 (CAdV-1) considering the two types of sampling methods in the 43 dogs (Wilcoxon signed-rank test).

When grouping the results into concordance categories and focusing exclusively on the 43 dogs sampled with the 21G lancet, the overall performance of the minimally invasive method showed further improvement. For nearly all pathogens, the good match values surpassed those obtained from the total cohort (*n* = 55). CAdV-1 achieved a perfect score with 100% concordance (vs. 96.4% in the total cohort), followed by CPV-2, which rose to 95.3% (vs. 94.5%). In contrast, the good match rate for CDV showed a slight numerical decrease to 90.7% (vs. 92.7% in the total cohort). This minor shift is a statistical consequence of the smaller sample size (*n* = 43), as the absolute number of dogs categorized as a bad match remained constant at four (4/43). As previously highlighted, these four cases represent the only instances where a significant discrepancy was maintained despite the use of the optimized 21G device, suggesting that factors other than sampling volume may influence the results in a limited subset of individuals. No statistically significant differences were observed between the two sampling methods for any of the pathogens evaluated, including the final 43 dogs (*p*-value 0.99 for CPV-2, 0.22 for CDV and 0.99 for CAdV-1), suggesting an almost complete agreement between the two methods, apart from CDV where the *p*-value remained above the threshold for statistical significance. Overall, the results demonstrate a high level of concordance between the two sampling techniques (Figure 8).

To evaluate the method’s maximum potential, a second Cohen’s kappa analysis was conducted on a refined cohort of 43 dogs, excluding dogs sampled with less suitable lancet types and focusing exclusively on the 21G lancet protocol (Appendix A Table A2). In this optimized group, the reliability of the micro-sampling technique further improved. For both CPV-2 and CAdV-1, the statistical analysis yielded a perfect agreement (kappa = 1.00), with 100% concordance between the two sampling sites. For CDV, the observed concordance remained very high at 95.3% (41/43). However, due to the limited number of non-protected dogs in this sub-population (only 1 identified by the gold standard), the Cohen’s kappa remained in the moderate range (0.48). These refined data confirm that, while the ear-prick method is highly accurate across all core pathogens, it is particularly robust when the 21G lancet is employed, effectively matching the diagnostic performance of traditional venous sampling for CPV-2 and CAdV-1.

### 3.4. Field and Clinical Application in a Real-World Outbreak

The ear-prick technique was performed ‘in series’ with exceptional efficiency. Adequate blood samples were successfully collected from all 70 puppies using a 21G lancet, with a 100% success rate despite the challenging environment. The technique allowed for the rapid collection of the volume required for immediate antibody titration without observable pain or the need for intensive restraint, even in subjects already compromised by environmental stress. These results confirm that the 1:2 dilution ear-prick method is a robust and humane alternative for large-scale screenings in sensitive populations, such as pediatric patients, especially those of small size.

The immunological screening provided clear diagnostic insights: while most puppies showed high Protective Antibody Titers (PATs) for CPV-2, three fell below the 1:80 threshold and were immediately vaccinated. In contrast, protection levels for CDV and CAdV-1 were inconsistent. Given the local epidemiological context and the unavailability of monovalent vaccines, health authorities prioritized the management of the CPV-2 risk, postponing further vaccination decisions to the puppies’ future clinicians. This outcome highlights the role of the ear-prick titration as a rapid decision-making tool in emergency clinical settings.

## 4. Discussion

Vaccination represents the most effective public health measure for the prevention and control of a wide range of dangerous, widespread, contagious, and often fatal diseases in both human and veterinary medicine. The so-called “core” vaccinations are those that are absolutely essential: they are generally recommended for all dogs and cats at least once in their lifetime to have a good chance of protection. However, vaccination is not synonymous with protection, and there are numerous factors that can influence the outcome of a vaccination, sometimes positively, much more often negatively. It is conceptually incorrect to assume that a dog or cat is definitely protected simply because it has been vaccinated [1,6,7,10,48].

This realization marks a fundamental shift toward personalized vaccination protocols, which represent one of the most significant advancements in modern small animal internal medicine. Immunization strategies are increasingly tailored to the specific needs of each patient by evaluating a complex set of variables, including, among other factors, the animal’s age, breed and size, overall health status and lifestyle. However, the cornerstone of this individualized approach is the feasibility of determining the actual immune protection of each dog. In this context, in-practice serological testing has emerged as an indispensable tool, allowing clinicians to move beyond general assumptions and make evidence-based decisions regarding the necessity of booster vaccinations for core pathogens (CPV-2, CDV, and CAdV-1) [1,2,6,7,34,40,45,46,55].

The adoption of in-clinic rapid tests to assess individual protection based on Protective Antibody Titers (PATs) is an approach increasingly embraced by veterinarians, breeders, and pet owners. This shift represents a conscious move away from non-personalized vaccination protocols, and it is strongly supported by international guidelines and immunology experts. For the antibody titration of core vaccine protection in dogs, the market offers several alternatives. Among these, VacciCheck^®^ Canine, used in this study, stands out as the only semi-quantitative dot ELISA-based test officially approved by multiple regulatory authorities worldwide and explicitly recommended by both WSAVA and AAHA [1,2]. In addition to ELISA-based systems, several immunochromatographic (lateral flow) tests are available, some providing visual results and others requiring dedicated digital readers. A fundamental common feature of all these point-of-care tests is the requirement for an extremely small blood sample (often only a few microliters). This technical characteristic makes minimally invasive blood collection not only an attractive option but also a scientifically viable and efficient alternative to traditional sampling. The classification of the ear-prick as a ‘minimally invasive’ technique is further supported by the tools employed. The 21G lancets used in this study are standard devices in human medicine for daily capillary blood sampling, such as glucose monitoring, where they are recognized for their high tolerability and minimal tissue trauma. This technical characteristic explains the lack of distress observed in our cohort; notably, in the pediatric group (*n* = 70), no puppies exhibited vocalization or withdrawal reflexes, often maintaining social behaviors like tail wagging during the procedure. The clinical evidence suggests that the ear-prick method represents a ‘fear-free’ alternative to venipuncture, significantly reducing the need for physical restraint and improving the overall welfare of the patient during routine monitoring. Furthermore, the selective use of the trichotomy only when strictly necessary (e.g., long and/or thick hair at the puncture site) personalizes the approach and ensures that the 1-person protocol remains as rapid as possible, particularly in large-scale screening scenarios.

The robustness of our proposal is evidenced by the high degree of overlap in the percentage distribution of protected versus non-protected dogs between the two sampling methods. As shown in the comparison of the optimized group (Table 5, see before), the ear-prick method yielded results that were virtually identical to standard venipuncture for all three core pathogens.

While capillary sampling may introduce minor variations in the blood-to-buffer ratio compared to precise laboratory pipetting, our data suggests that these fluctuations do not compromise the clinical classification. The clear cut-off levels of the semi-quantitative assay, combined with the high antibody titers typically found in the Italian canine population [48], provide a sufficient margin of safety to ensure that the ‘protected’ vs. ‘unprotected’ status remains consistent across sampling methods.

Our study highlights that the success of minimally invasive titration is strictly dependent on the choice of the appropriate tools. Initially, the use of smaller or less efficient lancets resulted in lower concordance rates; however, the transition to the 21G lancet proved to be the turning point. The 21G gauge was found to be the most reliable for obtaining an adequate blood volume in a single, minimally invasive step, ensuring a successful 1:2 dilution regardless of the patient’s dermatological characteristics. In fact, this specific gauge allowed for a more consistent and adequate blood flow, which is essential to provide the volume required by the VacciCheck^®^ test without excessive manipulation. This technical refinement led to a nearly 10 percentage point increase in full concordance, demonstrating that when performed with the correct instrumentation, the ear-prick method represents a viable alternative with comparable performance.

The absence of statistically significant differences between the two sampling methods suggests that they showed high diagnostic consistency for the assessment of antibody responses against all the three pathogens across both the full cohort and the reduced subset, supporting the robustness of the results.

Although minor discrepancies in antibody titers may occur between different testing methods, these are typically limited to a single dilution step and do not significantly affect the clinical classification of the animal’s immune status: particularly, the VacciCheck^®^ used in this study, a semi-quantitative dot-ELISA test, shows high sensitivity and specificity in identifying dogs with PATs, making it a reliable tool for determining the need for revaccination in clinical practice, regardless of minor numerical titer variations [56,57]. Moreover, the Cohen’s kappa coefficient serves as the final confirmation of the strong categorical agreement between the two sampling methods, ensuring that the clinical interpretation and the subsequent medical decision remain unchanged regardless of the sampling site: this means that, regardless of minor numerical fluctuations in titers, the veterinarian’s decision—to vaccinate or to postpone—remains consistently correct. The lower kappa value for CDV compared to the other two viruses would be primarily attributable to the high prevalence of protected individuals in the population, where even a few clinical discrepancies (4 dogs in this cohort) significantly penalize the coefficient. This discrepancy is likely also attributable to the inherent characteristics of the CDV titration kit, which appears more sensitive to minimal variations in sample volume or the presence of initial micro-clotting, factors that are more frequent in capillary blood compared to venous samples. However, it is crucial to note that all discordant cases were ‘conservative’: the ear-prick method never overestimated the antibody titer compared to venipuncture. From a clinical perspective, this ensures that the risk of misclassifying an unprotected dog as protected is effectively nullified, maintaining the safety and reliability of the test for field screening.

Furthermore, according to the manufacturer’s instructions and the established analytical tolerance of the assay, a single titer variation is considered clinically irrelevant and does not alter the overall interpretation of the immune status. Finally, the 2024 WSAVA vaccination guidelines [1] state that the mere presence of antibodies, regardless of the titer, indicates protection, but the clinical relevance of antibody quantification remains a topic of scientific interest. In our study, we observed that discrepancies between classical and capillary sampling in many cases were minimal (±1 titer). This high level of numerical concordance is crucial for two reasons: first, it ensures that the clinical decision (to vaccinate or not) remains identical regardless of the sampling method, in full compliance with current guidelines. Second, it demonstrates that the ear-prick technique maintains the semi-quantitative resolution required to assess the robustness of the immune response, as suggested by the correlation between higher titers and stronger protection as suggested in the 2016 WSAVA vaccination guidelines [58].

Based on the high feasibility and accuracy observed in our study, several promising clinical applications emerge. For geriatric patients, often affected by chronic conditions and fragile vasculature, the ear-prick method could offer a less invasive alternative for routine monitoring, counteracting the common misconception among owners that elderly dogs no longer require protection or are “too old” for further vaccinations. Similarly, in shelter medicine, the speed and cost-effectiveness of this technique could significantly improve large-scale screening protocols. Further studies that objectively assess the usefulness of this alternative sampling method (e.g., pain or restraint scales) would be helpful in quantifying these benefits.

The practical implications of our findings are best illustrated by the field application conducted during the judicial seizure. This real-world application underscores that the ear-prick technique is not merely a laboratory refinement but a crucial tool for emergency immunological assessment. In high-pressure environments, such as shelter or forensic medicine, the ability to obtain reliable antibody titers without the logistical and physical burden of traditional venipuncture represents a significant advancement. The success observed in the 70-puppy cohort demonstrates that this method effectively bridges the gap between analytical precision and the immediate needs of sanitary crises, ensuring that animal welfare remains a priority even during large-scale screenings. Furthermore, the rapid decision-making enabled by this technique, allowing for immediate, targeted vaccinations, highlights its potential to optimize infectious disease management in vulnerable populations.

### 4.1. Limitation of the Study

While the results of this study are highly encouraging, some limitations must be acknowledged. First, the sample size, particularly for the optimized 21G lancet group (*n* = 43 dogs), represents a relatively small cohort. Although statistically sufficient for this comparison, future research involving a larger and more diverse population of dogs, varying in breed, age, and coat type, is necessary to further confirm the universal applicability of the ear-prick technique.

A second consideration involves the 1:2 dilution of the capillary sample. In field settings, the use of precision micropipettes is often impractical; thus, the blood volume is diluted ‘by eye’ with sterile distilled water. While this introduces a degree of approximation, it does not undermine the clinical validity of the test. From an immunological perspective, this approximation acts as a ‘safety buffer’. Any deviation from the exact 1:2 ratio would likely lead to a slight underestimation of the antibody titer, potentially classifying a borderline dog as ‘non-protective’. Crucially, the opposite scenario (a false positive where an unprotected dog is incorrectly classified as protected) is mathematically impossible, as dilution cannot amplify sub-threshold antibody concentrations. This conservative bias aligns with the core principle of preventive medicine: prioritizing maximum population immunity and individual safety over absolute analytical precision.

The sample size, particularly in the subgroup analysis, may have limited the statistical power to detect small differences. Additionally, biological variability among animals may have contributed to the observed dispersion of values. The sample size of the 21G group (*n* = 43) also represents a limitation for the detection of rare ‘non-responder’ individuals, a challenge common to any sampling method in similar-sized cohorts. However, since the study aimed to evaluate the agreement between the ear-prick and venipuncture, the results are sufficient to show that both techniques provide consistent diagnostic information. This preliminary comparison suggests that the method is reliable for routine clinical use, though larger populations will be necessary to further characterize its performance in rare clinical scenarios.

A potential limitation of this study is the small number of truly unprotected animals within the cohort, particularly for CPV-2 and CDV. This distribution resulted in an unstable kappa statistic for these specific pathogens. However, it is important to note that this low frequency of seronegative subjects is directly consistent with the current Italian epidemiological scenario. Recent large-scale surveillance on 1027 dogs in Italy [48] has documented exceptionally high seroprevalence rates, with protection levels reaching 90.8% for CPV-2. Therefore, the demographic composition of our study group, while limiting for certain statistical indices, represents a faithful and representative reflection of the high herd immunity and natural boosting effects characteristic of the national canine population.

Another practical limitation encountered during the initial sampling phase of this study was the rapid coagulation of blood within the standard non-heparinized glass capillary tubes. Due to the small diameter of the capillary and the time required to collect the drop from the ear surface, the blood occasionally clotted before it could be effectively transferred to the test tube. This technical hurdle was successfully resolved in subsequent samplings, conducted after the completion of this study, by utilizing heparinized micro-hematocrit capillaries. The presence of an anticoagulant coating within the capillary has prevented premature clotting and has ensured a fluid, seamless transfer of the sample. This refinement has significantly improved the efficiency of the workflow, and the use of this kind of capillary is suggested as the standard protocol for future implementations of the ear-prick technique.

Finally, the CDV spots on the VacciCheck^®^ scale often exhibit lower color intensity, which can complicate the comparative reading between classical and capillary samples, especially when titers are near the threshold. From a biological perspective, unlike the more environmentally resilient CPV-2 and CAdV-1, the Canine Distemper Virus is an enveloped and labile pathogen. The lack of frequent natural environmental boosting, due to its requirement for direct contact transmission, often results in lower circulating antibody levels over time. While these minor discrepancies (±1 titer) do not impair the overall clinical interpretation of the patient’s immune status, they highlight a known intrinsic challenge in the semi-quantitative assessment of CDV antibodies, a phenomenon also noted by other authors [48,50,59,60,61].

### 4.2. Future Perspectives

While other anatomical sites, such as the inner labial mucosa, have been suggested for capillary sampling (similar to techniques used for blood typing), we believe this approach presents significant drawbacks for antibody titration. First, the presence of saliva poses a high risk of uncontrolled sample dilution, which would further compromise the accuracy of the 1:2 ratio and the subsequent clinical interpretation of the actual antibody titer. Furthermore, from a behavioral perspective, accessing the inner lip requires a degree of physical restraint that is often poorly tolerated, especially by feline patients. This could trigger defensive reactions and increase the risk of painful stimuli, counteracting the primary goal of a ‘low-stress’ procedure.

Therefore, our ongoing research continues to prioritize the ear-prick method and is currently exploring its application in feline patients. Interestingly, we are also evaluating the use of paw pads as an alternative site in cats. While canine paw pads were found to be too thick and keratinized for effective lancet penetration, the feline pad tissue offers a viable alternative due to its different histological characteristics. This approach offers the distinct advantage of easier physical restraint, as many cats are more comfortable with limb handling than with facial manipulation. Moreover, this technique aligns with established clinical practices already familiar to practitioners, such as capillary glucose monitoring in diabetic feline patients.

Finally, future studies will investigate the use of innovative, ready-to-use capillary collection devices that integrate blood collection and anticoagulation into a single workflow. While these devices are promising, their current design, optimized for larger human peripheral samplings, would require significant adaptation to the much smaller volumes (5–10 µL) collected during minimally invasive pet sampling.

## 5. Conclusions

The use of in-clinic rapid tests to assess individual protection based on Protective Antibody Titers (PATs) is an approach increasingly adopted by veterinarians, breeders, and pet owners as a way to move away from blind vaccination or routine vaccination carried out in the same manner every time, and it is widely supported by all international vaccination guidelines and field experts. A common feature of all these tests (ELISA and lateral flow) is the requirement for a very small blood sample (on the order of a few microliters), making minimally invasive blood collection an attractive and viable option.

The present study demonstrates that the 21G lancet ear-prick method coupled with a 1:2 capillary dilution is a highly reliable and technically sound alternative to traditional venous sampling for the assessment of protective antibodies in dogs. Our results confirmed a very high correlation with the gold standard sampling method, ensuring that no false-positive results occur due to the minimal dilution required. Beyond its analytical accuracy, this micro-sampling approach represents a significant advancement in veterinary medical ethics and animal welfare. By minimizing restraint and physical pain, the ear-prick technique reduces the stress associated with clinical visits and facilitates the implementation of personalized vaccination protocols in daily practice. Furthermore, as demonstrated by the large-scale application in a high-risk forensic setting involving 70 puppies, this method is uniquely suited for emergency management and collective disease surveillance. Its logistical simplicity and rapid turnaround time allow for immediate, evidence-based clinical decisions without compromising animal wellbeing.

In conclusion, the adoption of this minimally invasive technique empowers veterinarians to transition from a “one-size-fits-all” vaccination strategy to a precise, welfare-oriented immunological monitoring, ultimately enhancing both individual patient care and public health standards. From a practical perspective, the demonstrated interchangeability of the two sampling methods represents a significant advantage in clinical settings, as it allows the choice of technique to be guided by considerations such as ease of use, operator preference, cost, and, importantly, animal welfare, rather than analytical performance.

While these preliminary results demonstrate a strong clinical agreement between the conventional and the minimally invasive sampling methods, further prospective studies on larger independent populations are warranted to definitively establish the method’s performance across different clinical settings.

## Figures and Tables

**Table 1:** Concordance percentages between antibody titers obtained from standard venipuncture (A) and minimally invasive sampling (B) across the 55 dogs enrolled in the study. Minimally invasive sampling results were multiplied by the dilution factor (1:2).

	% (No. of Dogs)		
Match A vs. B	CPV-2	CDV	CAdV-1
Full match	72.7% (40/55)	70.9% (39/55)	65.5% (36/55)
+½ titer	0.0% (0/55)	0.0% (0/55)	0.0% (0/55)
−½ titer	0.0% (0/55)	0.0% (0/55)	0.0% (0/55)
+1 titer	14.6% (8/55)	12.7% (7/55)	20.0% (11/55)
−1 titer	7.3% (4/55)	9.1% (5/55)	10.9% (6/55)
+1 ½ titer	0.0% (0/55)	0.0% (0/55)	0.0% (0/55)
−1 ½ titer	0.0% (0/55)	0.0% (0/55)	0.0% (0/55)
+2 titer	1.8% (1/55)	3.7% (2/55)	0.0% (0/55)
−2 titer	1.8% (1/55)	1.8% (1/55)	0.0% (0/55)
+3 titer	1.8% (1/55)	1.8% (1/55)	3.6% (2/55)
−3 titer	0.0% (0/55)	0.0% (0/55)	0.0% (0/55)
TOTAL	100% (55/55)	100% (55/55)	100% (55/55)

White background: good match (full match or ±1 titer difference); light grey: poor match (±1 ½ titer difference); dark grey: bad match (±2 or more titer difference). Legend: the plus sign “+” (e.g., “+1, +1 ½, +2”) indicates that the titer from standard sampling was higher than the minimally invasive one (the number indicates the magnitude of the difference). Similarly, the negative sign “−” (e.g., “−1, −1 ½, −2”) indicates that the standard sampling titer was lower than the minimally invasive one.

**Table 2:** Concordance between antibody titers from standard venipuncture (A) and minimally invasive sampling (B), categorized by agreement level across the 55 dogs enrolled in the study. Minimally invasive sampling results were multiplied by the dilution factor (1:2).

	% (No. of Dogs)		
Match A vs. B	CPV-2	CDV	CAdV-1
GOOD (equal, ±½ titer, ±1 titer)	94.5% (52/55)	92.7% (51/55)	96.4% (53/55)
POOR (±1 ½ titer)	0.0% (0/55)	0.0% (0/55)	0.0% (0/55)
BAD (±2 titers, ±3 titers)	5.5% (3/55)	7.3% (4/55)	3.6% (2/55)
TOTAL	100% (55/55)	100% (55/55)	100% (55/55)

White background: good match (full match or ±1 titer difference); light grey: poor match (±1 ½ titer difference); dark grey: bad match (±2 or more titer difference).

**Table 3:** Number of dogs categorized by the needle gauge used for minimally invasive sampling.

Needle	% (No. of Dogs)
21G	78.2% (43/55)
28G	21.8% (12/55)
TOTAL	100% (55/55)

**Table 4:** Concordance percentages between antibody titers obtained from standard venipuncture (A) and minimally invasive sampling (B) for the 43 dogs sampled with 21G lancets. Minimally invasive sampling results were multiplied by the dilution factor (1:2).

	% (No. of Dogs)		
Match A vs. B	CPV-2	CDV	CAdV-1
Full match	81.4% (35/43)	76.8% (33/43)	74.4% (32/43)
+½ titer	0.0% (0/43)	0.0% (0/43)	0.0% (0/43)
−½ titer	0.0% (0/43)	0.0% (0/43)	0.0% (0/43)
+1 titer	7.0% (3/43)	4.7% (2/43)	14.0% (6/43)
−1 titer	7.0% (3/43)	9.2% (4/43)	11.6% (5/43)
+1 ½ titer	0.0% (0/43)	0.0% (0/43)	0.0% (0/43)
−1 ½ titer	0.0% (0/43)	0.0% (0/43)	0.0% (0/43)
+2 titer	0.0% (0/43)	4.7% (2/43)	0.0% (0/43)
−2 titer	2.3% (1/43)	2.3% (1/43)	0.0% (0/43)
+3 titer	2.3% (1/43)	2.3% (1/43)	0.0% (0/43)
−3 titer	0.0% (0/43)	0.0% (0/43)	0.0% (0/43)
TOTAL	100% (43/43)	100% (43/43)	100% (43/43)

White background: good match (full match or ±1 titer difference); light grey: poor match (±1 ½ titer difference); dark grey: bad match (±2 or more titer difference). Legend: the plus sign “+” (e.g., “+1, +1 ½, +2”) indicates that the titer from standard sampling was higher than the minimally invasive one (the number indicates the magnitude of the difference). Similarly, the negative sign “−” (e.g., “−1, −1 ½, −2”) indicates that the standard sampling titer was lower than the minimally invasive one.

**Table 5:** Concordance between antibody titers from standard venipuncture (A) and minimally invasive sampling (B), categorized by agreement level for the 43 dogs sampled with 21G lancets. Minimally invasive sampling results were multiplied by the dilution factor (1:2).

	% (No. of Dogs)		
Match A vs. B	CPV-2	CDV	CAdV-1
GOOD (equal, ±½ titer, ±1 titer)	95.3% (41/43)	90.7% (39/43)	100.0% (43/43)
POOR (±1 ½ titer)	0.0% (0/43)	0.0% (0/43)	0.0% (0/43)
BAD (±2 titers, ±3 titers)	4.7% (2/43)	9.3% (4/43)	0.0% (0/43)
TOTAL	100% (43/43)	100% (43/43)	100% (43/43)

White background: good match (full match or ±1 titer difference); light grey: poor match (±1 ½ titer difference); dark grey: bad match (±2 or more titer difference).

## Data Availability

The authors confirm that the datasets analyzed during the study are available from the first author or the corresponding author upon reasonable request.

## Appendix A

**Table A1:** VacciCheck^®^ Canine: correspondence between S scale units and antibody titers, sensitivity, and specificity for Canine Parvovirus type 2 (CPV-2), Canine Distemper Virus (CDV), and Canine Adenovirus type 1 (CAdV-1).

	CPV (%)	CDV (%)	CAV (%)
S0	<1:20	<1:8	<1:4
S1	1:20	1:8	1:4
S2	1:40	1:16	1:8
**S3 (threshold)**	**1:80**	**1:32**	**1:16**
S4	1:160	1:64	1:32
S5	1:320	1:128	1:64
S6	1:640	1:256	1:128
>S6	>1:640	>1:256	>1:128
Sensitivity (%)	88	100	94
Specificity (%)	100	92	93

**Table A2:** Cohen’s K for the agreement between the two sampling methods.

K Value	Agreement		
0.81–1.00	Almost perfect		
0.61–0.80	Substantial		
0.41–0.60	Moderate		
0.21–0.40	Fair		
0.01–0.20	Slight		
<0.01	Poor		
**1. Population of 55 dogs**			
CPV-2—55 dogs	Minimally invasive Protected	Minimally invasive Unprotected	**TOTAL** **Classic**
Classical Protected	54	0	**54**
Classical Unprotected	0	1	**1**
**TOTAL Minimally invasive**	**54**	**1**	**55**
Cohen’s K = 1.00—**Agreement: Almost perfect**			
CDV—55 dogs	Minimally invasive Protected	Minimally invasive Unprotected	**TOTAL** **Classic**
Classical Protected	49	4	**53**
Classical Unprotected	0	2	**2**
**TOTAL Minimally invasive**	**49**	**6**	**55**
Cohen’s K = 0.471—**Agreement: Moderate**			
CAdV-1—55 dogs	Minimally invasive Protected	Minimally invasive Unprotected	**TOTAL** **Classic**
Classical Protected	40	2	**42**
Classical Unprotected	0	13	**13**
**TOTAL Minimally invasive**	**40**	**15**	**55**
Cohen’s K = 0.904—**Agreement: Almost perfect**			
**2. Population of 43 dogs**			
CPV-2—43 dogs	Minimally invasive Protected	Minimally invasive Unprotected	**TOTAL** **Classic**
Classical Protected	42	0	**42**
Classical Unprotected	0	1	**1**
**TOTAL Minimally invasive**	**42**	**1**	**43**
Cohen’s K = 1.00—**Agreement: Almost perfect**			
CDV—43 dogs	Minimally invasive Protected	Minimally invasive Unprotected	**TOTAL** **Classic**
Classical Protected	40	2	**42**
Classical Unprotected	0	1	**1**
**TOTAL Minimally invasive**	**40**	**3**	**43**
Cohen’s K = 0.482—**Agreement: Moderate**			
CAdV-1—43 dogs	Minimally invasive Protected	Minimally invasive Unprotected	**TOTAL** **Classic**
Classical Protected	31	0	**31**
Classical Unprotected	0	12	**12**
**TOTAL Minimally invasive**	**31**	**12**	**43**
Cohen’s K = 1.00—**Agreement: Almost perfect**			

## References

[1] Squires R.A., Crawford C., Marcondes M., Whitley N. (2024). 2024 Guidelines for the Vaccination of Dogs and Cats—Compiled by the Vaccination Guidelines Group (VGG) of the World Small Animal Veterinary Association (WSAVA). J. Small Anim. Pract..

[2] Ellis J., Marziani E., Aziz C., Brown C.M., Cohn L.A., Lea C., Moore G.E., Taneja N. (2022). 2022 AAHA Canine Vaccination Guidelines. J. Am. Anim. Hosp. Assoc..

[3] Australian Veterinary Association (AVA) Vaccination of Dogs and Cats. https://www.ava.com.au/policy-advocacy/policies/companion-animals-health/vaccination-of-dogs-and-cats/.

[4] British Veterinary Association (BVA) Getting Your Pet Vaccinated. https://www.bva.co.uk/media/2649/client_leaflet_9_-_getting_your_pet_vaccinated.pdf.

[5] Joint American Veterinary Medical Association (AVMA)-Federation of Veterinarians of Europe (FVE)-Canadian Veterinary Medical Association (CVMA) Statement on the Benefits of Animal Vaccination Programs in Advancing Animal and Human Health. https://fve.org/cms/wp-content/uploads/AVMA-CVMA-FVE_vacconation_joint-paper.docx.pdf.

[6] Dall’Ara P. (2024). Vaccini e Vaccinazioni Degli Animali Da Compagnia.

[7] Altman K.D., Kelman M., Ward M.P. (2017). Are Vaccine Strain, Type or Administration Protocol Risk Factors for Canine Parvovirus Vaccine Failure?. Vet. Microbiol..

[8] Zhou P., Zeng W., Zhang X., Li S. (2017). The Genetic Evolution of Canine Parvovirus—A New Perspective. PLoS ONE.

[9] Decaro N., Buonavoglia C. (2012). Canine Parvovirus—A Review of Epidemiological and Diagnostic Aspects, with Emphasis on Type 2c. Vet. Microbiol..

[10] Decaro N., Buonavoglia C., Barrs V.R. (2020). Canine Parvovirus Vaccination and Immunisation Failures: Are We Far from Disease Eradication?. Vet. Microbiol..

[11] Mila H., Grellet A., Feugier A., Desario C., Decaro N., Buonavoglia C., Mariani C., Chastant-Maillard S. (2018). General and Type 2 Parvovirus-Specific Passive Immune Transfer in Puppies—Evaluation by Early Growth. Reprod. Domest. Anim..

[12] Dall’Ara P., Lauzi S., Filipe J., Caseri R., Beccaglia M., Desario C., Cavalli A., Aiudi G.G., Buonavoglia C., Decaro N. (2021). Discrepancy Between In-Clinic and Haemagglutination-Inhibition Tests in Detecting Maternally-Derived Antibodies Against Canine Parvovirus in Puppies. Front. Vet. Sci..

[13] Decaro N., Crescenzo G., Desario C., Cavalli A., Losurdo M., Colaianni M.L., Ventrella G., Rizzi S., Aulicino S., Lucente M.S. (2014). Long-Term Viremia and Fecal Shedding in Pups after Modified-Live Canine Parvovirus Vaccination. Vaccine.

[14] Hussain K., Khan Y., Ullah Q., Rabbani A.H., Naseer O., Raza A., Shahid M., Ali S., Ali A. (2021). Canine Parvo Virus: A Review on Current Perspectives in Seroprevalence, Diagnostics and Therapeutics. Glob. Vet..

[15] Sykes J. (2022). Greene’s Infectious Diseases of the Dog and Cat.

[16] Duque-Valencia J., Sarute N., Olarte-Castillo X.A., Ruíz-Sáenz J. (2019). Evolution and Interspecies Transmission of Canine Distemper Virus—An Outlook of the Diverse Evolutionary Landscapes of a Multi-Host Virus. Viruses.

[17] Sykes J., Vandevelde M., Sykes J.E. (2022). Canine Distemper Virus Infection. Greene’s Infectious Diseases of the Dog and Cat.

[18] Meazzi S., Filipe J., Fiore A., Di Bella S., Mira F., Dall’Ara P. (2022). Agreement between In-Clinics and Virus Neutralization Tests in Detecting Antibodies against Canine Distemper Virus (CDV). Viruses.

[19] Mira F., Purpari G., Di Bella S., Vicari D., Schirò G., Di Marco P., Macaluso G., Battilani M., Guercio A. (2018). Update on Canine Distemper Virus (CDV) Strains of Arctic-like Lineage Detected in Dogs in Italy. Vet. Ital..

[20] Trogu T., Canziani S., Salvato S., Bianchi A., Bertoletti I., Gibelli L.R., Alborali G.L., Barbieri I., Gaffuri A., Sala G. (2021). Canine Distemper Outbreaks in Wild Carnivores in Northern Italy. Viruses.

[21] Martella V., Elia G., Buonavoglia C. (2008). Canine Distemper Virus. Vet. Clin. N. Am. Small Anim. Pract..

[22] Decaro N., Campolo M., Elia G., Buonavoglia D., Colaianni M.L., Lorusso A., Mari V., Buonavoglia C. (2007). Infectious Canine Hepatitis: An “Old” Disease Reemerging in Italy. Res. Vet. Sci..

[23] Decaro N., Sykes J.E. (2022). Infectious Canine Hepatitis and Feline Adenovirus Infection. Greene’s Infectious Diseases of the Dog and Cat.

[24] Decaro N., Martella V., Buonavoglia C. (2008). Canine Adenoviruses and Herpesvirus. Vet. Clin. N. Am. Small Anim. Pract..

[25] Dowgier G., Lahoreau J., Lanave G., Losurdo M., Varello K., Lucente M.S., Ventriglia G., Bozzetta E., Martella V., Buonavoglia C. (2018). Sequential Circulation of Canine Adenoviruses 1 and 2 in Captive Wild Carnivores, France. Vet. Microbiol..

[26] Mira F., Puleio R., Schirò G., Condorelli L., Di Bella S., Chiaramonte G., Purpari G., Cannella V., Balboni A., Randazzo V. (2022). Study on the Canine Adenovirus Type 1 (CAdV-1) Infection in Domestic Dogs in Southern Italy. Pathogens.

[27] Schultz R.D. (2006). Duration of Immunity for Canine and Feline Vaccines: A Review. Vet. Microbiol..

[28] Schultz R.D., Thiel B., Mukhtar E., Sharp P., Larson L.J. (2010). Age and Long-Term Protective Immunity in Dogs and Cats. J. Comp. Pathol..

[29] Möstl K. (2016). Duration of Vaccine-Induced Immunity. EJCAP.

[30] Chastant-Maillard S., Freyburger L., Marcheteau E., Thoumire S., Ravier J., Reynaud K. (2012). Timing of the Intestinal Barrier Closure in Puppies. Reprod. Domest. Anim..

[31] Chastant-Maillard S., Mila H. (2016). Canine Colostrum. Vet. Focus.

[32] Mila H., Grellet A., Mariani C., Feugier A., Guard B., Suchodolski J., Steiner J., Chastant-Maillard S. (2017). Natural and Artificial Hyperimmune Solutions: Impact on Health in Puppies. Reprod. Domest. Anim..

[33] Mila H., Feugier A., Grellet A., Anne J., Gonnier M., Martin M., Rossig L., Chastant-Maillard S. (2015). Immunoglobulin G Concentration in Canine Colostrum: Evaluation and Variability. J. Reprod. Immunol..

[34] Mila H., Feugier A., Grellet A., Anne J., Gonnier M., Martin M., Rossig L., Chastant-Maillard S. (2014). Inadequate Passive Immune Transfer in Puppies: Definition, Risk Factors and Prevention in a Large Multi-Breed Kennel. Prev. Vet. Med..

[35] Mugnier A., Chastant S., Saegerman C., Gaillard V., Grellet A., Mila H. (2021). Management of Low Birth Weight in Canine and Feline Species: Breeder Profiling. Animals.

[36] Chastant-Maillard S., Aggouni C., Albaret A., Fournier A., Mila H. (2017). Canine and Feline Colostrum. Reprod. Domest. Anim..

[37] Chastant S., Mila H. (2019). Passive Immune Transfer in Puppies. Anim. Reprod. Sci..

[38] Belizário J.E., Garay-Malpartida M. (2023). Growth Hormone, Immunosenescence and Vaccination Failure in the Elderly. Clin. Immunol. Commun..

[39] Pereira M., Valério-Bolas A., Saraiva-Marques C., Alexandre-Pires G., Pereira da Fonseca I., Santos-Gomes G. (2019). Development of Dog Immune System: From in Uterus to Elderly. Vet. Sci..

[40] Dall’Ara P., Lauzi S., Turin L., Castaldelli G., Servida F., Filipe J. (2023). Effect of Aging on the Immune Response to Core Vaccines in Senior and Geriatric Dogs. Vet. Sci..

[41] Fulop T., Larbi A., Dupuis G., Le Page A., Frost E.H., Cohen A.A., Witkowski J.M., Franceschi C. (2018). Immunosenescence and Inflamm-Aging As Two Sides of the Same Coin: Friends or Foes?. Front. Immunol..

[42] McKenzie B.A. (2022). Comparative Veterinary Geroscience: Mechanism of Molecular, Cellular, and Tissue Aging in Humans, Laboratory Animal Models, and Companion Dogs and Cats. Am. J. Vet. Res..

[43] Mitchell S., Zwijnenberg R., Huang J., Hodge A., Day M. (2012). Duration of Serological Response to Canine Parvovirus-Type 2, Canine Distemper Virus, Canine Adenovirus Type 1 and Canine Parainfluenza Virus in Client-Owned Dogs in Australia. Aust. Vet. J..

[44] Dodds W.J. (2021). Early Life Vaccination of Companion Animal Pets. Vaccines.

[45] Rashid A., Rasheed K., Akhtar M. (2009). Factors Influencing Vaccine Efficacy—A General Review. J. Anim. Plant Sci..

[46] DiGangi B.A., Dingman P.A., Grijalva C.J., Belyeu M., Tucker S., Isaza R. (2019). Prevalence and Risk Factors for the Presence of Serum Antibodies against Canine Distemper, Canine Parvovirus, and Canine Adenovirus in Communities in Mainland Ecuador. Vet. Immunol. Immunopathol..

[47] Dall’Ara P., Filipe J., Pilastro C., Turin L., Lauzi S., Gariboldi E.M., Stefanello D. (2023). Can Chemotherapy Negatively Affect the Specific Antibody Response toward Core Vaccines in Canine Cancer Patients?. Vet. Sci..

[48] Dall’Ara P., Lauzi S., Zambarbieri J., Servida F., Barbieri L., Rosenthal R., Turin L., Scarparo E., Filipe J. (2023). Prevalence of Serum Antibody Titers against Core Vaccine Antigens in Italian Dogs. Life.

[49] Roth J.A., Spickler A.R. (2010). Duration of Immunity Induced by Companion Animal Vaccines. Anim. Health Res. Rev..

[50] Egerer A., Schaefer Z., Larson L. (2022). A Point-of-Care Dot Blot ELISA Assay for Detection of Protective Antibody against Canine Adenovirus, Canine Parvovirus, and Canine Distemper Virus Is Diagnostically Accurate. J. Am. Vet. Med. Assoc..

[51] Janowitz L., Abd El Wahed A., Truyen U., Hofmann-Lehmann R., Spiri A.M. (2025). Antibody Titer Testing in Dogs: Evaluation of Three Point-of-Care Tests for Canine Core Vaccine Antigens Compared to Virus Neutralization. Vet. Sci..

[52] Salomon K., de Lange T., Calis A., Radier O., Krosse J. (2022). In-Clinic Canine IgG Antibody Titer Test Comparative Study: Results from Five Clinics. Isr. J. Vet. Med..

[53] Waner T., Mazar S., Keren-Kornblatt E. (2006). Application of a Dot Enzyme-Linked Immunosorbent Assay for Evaluation of the Immune to Canine Parvovirus and Distemper Virus in Adult Dogs before Revaccination. J. Vet. Diagn. Investig..

[54] Biogal Galed Labs. Acs. Ltd. VacciCheck^®^ Titer Testing. https://www.biogal.com/products/vaccicheck/.

[55] Killey R., Mynors C., Pearce R., Nell A., Prentis A., Day M.J. (2018). Long-Lived Immunity to Canine Core Vaccine Antigens in UK Dogs as Assessed by an in-Practice Test Kit. J. Small Anim. Pract..

[56] Mende K., Stuetzer B., Truyen U., Hartmann K. (2014). Evaluation of an In-House Dot Enzyme-Linked Immunosorbent Assay to Detect Antibodies against Feline Panleukopenia Virus. J. Feline Med. Surg..

[57] Gray L.K., Crawford P.C., Levy J.K., Dubovi E.J. (2012). Comparison of Two Assays for Detection of Antibodies against Canine Parvovirus and Canine Distemper Virus in Dogs Admitted to a Florida Animal Shelter. J. Am. Vet. Med. Assoc..

[58] Day M.J., Horzinek M.C., Schultz R.D., Squires R.A. (2016). WSAVA Guidelines for the Vaccination of Dogs and Cats. J. Small Anim. Pract..

[59] Litster A., Nichols J., Volpe A. (2012). Prevalence of Positive Antibody Test Results for Canine Parvovirus (CPV) and Canine Distemper Virus (CDV) and Response to Modified Live Vaccination against CPV and CDV in Dogs Entering Animal Shelters. Vet. Microbiol..

[60] Litster A.L., Pressler B., Volpe A., Dubovi E. (2012). Accuracy of a Point-of-Care ELISA Test Kit for Predicting the Presence of Protective Canine Parvovirus and Canine Distemper Virus Antibody Concentrations in Dogs. Vet. J..

[61] Lechner E.S., Crawford P.C., Levy J.K., Edinboro C.H., Dubovi E.J., Caligiuri R. (2010). Prevalence of Protective Antibody Titers for Canine Distemper Virus and Canine Parvovirus in Dogs Entering a Florida Animal Shelter. J. Am. Vet. Med. Assoc..

